# Remarks on the Ill Consequences Arising from Unskilful Venæsection

**Published:** 1799-11

**Authors:** John Ring

**Affiliations:** New Street, Hanover Square


### *Remarks on the ill Consequences arising from unskilful Venæsection; by* Mr. Ring, *Member of the Corporation of Surgeons.*

---

### *To the Editors of the Medical and Physical Journal.*

Gentlemen,

In your last number appeared some Observations by Mr. Pulley, of Bedford, on the Case of ill Effects produced by Venæsection, published by Dr. Vaughan, of Rochester; by which we learn, that Mr. P. agrees with me in rejecting the notion of a morbid poison; but not in the opinion, that the melancholy catastrophe was occasioned by the length of the orifice, and its not being properly closed.

Mr. P. thinks the inflammation which proved fatal, was owing to a peculiarity of constitution; which, he says, you may call irritability if you please. But, unfortunately for his argument, the man who lost his life on this occasion, is described by the learned gentleman who drew up the case, to have been athletic, in the prime of life; and from the opinion given by his surgeon previous to the operation, we may presume he was then in full health and vigour.

As Mr. P. alludes, with due respect to that celebrated anatomist and physiologist Mr. Hunter, I shall appeal to the same authority. He says, in his Treatise on the Blood, Inflammation, &c. p. 230: "A wound made upon a person of a healthy constitution, and sound parts, will unite almost at once." This destroys every idea of an irritability, or any other manifest or occult quality, existing in a robust constitution, that can tend to prevent or retard union by the first intention.

It may then be asked, what impeded this salutary process in the case under consideration? To this I answer, The incision was *very large*; and, when a surgeon was called in, the sides of the vein appeared to him not united, but inflamed and suppurated. In addition to the natural difficulty of keeping the lips of the wound in perfect contact, when it is immoderately long, its situation in the bend of the elbow is an impediment; for as often as the arm is put into a state of flexion, the orifice will have a tendency to open, in proportion to its length.

This

This opinion is not founded upon mere theory; I have seen repeated instances of considerable inflammation and suppuration, evidently arising from that cause.

Mr. P. doubts the propriety of Dr. VAUGHAN's decision; and says, justice ought to be done to the barber.—I hope no one will rashly condemn the barber;

*For a barber to flay is a barbarous deed,*

as the author of Salmagundi rightly observes. At the same time, I think Mr. P. rather over-rates the chirurgical skill of the barber. He says, he firmly believes, that if the surgeon had bled him himself, the same would have been the issue. If this opinion can be justified, it may prove a considerable saving to the public in these hard times; for it must be unnecessary to employ a surgeon, in cases requiring venæsection, if it can be performed full as well by a barber.

Having bled, and seen bled, numbers of persons endowed with every degree of irritability, I cannot recollect a single instance of any serious ill consequence arising from that cause; but I have known, on various occasions, the habit of the patient blamed by the surgeon, when the patient had much greater reason to blame the surgeon for his want of skill.

Mr. P. agrees with me, that in all probability the mischief was not produced by any poisonous matter. He thinks, as I do, that "the symptoms of such cases were erroneously considered, till the keen scrutinizing knife of Mr. HUNTER exhibited the disease in its proper colour."—If Mr. P. will allow the edge of Mr. HUNTER's wit to have been as keen as that of his knife, and that he was any judge of those cases to which he paid such particular attention, I shall convince him that his own opinion also is erroneous.

Mr. P. says, "It has been imagined, the inflammation has been induced by the external orifice not being effectually closed; but this idea is by no means correct." What says Mr. HUNTER? After observing that some have ascribed the bad consequences arising from venæsection, to the puncture of a nerve, aponeurosis, or tendon, he adds, "By a third set of physiologists, the constitution has been blamed; and the symptoms have been said to arise *only in bad habits*. But here also experience is on the opposite side; and a person who has before enjoyed the most perfect health, is just as liable to the accidents from bleeding, as one of a weakened or crazy constitution. And it may even be observed, that, where such accidents have happened, the patient has been bled in the opposite arm, without any inconvenience."

In

In the third volume of the Medical Commentaries, from which I extracted the foregoing paragraph, Dr. DUNCAN informs us, that for various reaſons which he ſpecifies, Mr. HUNTER concluded, that the miſchief aroſe from an inflammation of the cavity of the vein. In a fatal caſe there mentioned, the orifice was, as in Dr. V's. caſe, ſtill open.

" Mr. HUNTER is diſpoſed to think," ſays Dr. DUNCAN, " that the principal cauſe producing the inflammation of a vein after bleeding, is the want of diſpoſition to heal. This may at firſt ariſe either from its being expoſed, or in conſequence of the lips of the orifice in the ſkin not being properly brought together. If an injury be done to one part of a cavity, the ſtimulus, which ariſes from its being an imperfect cavity, goes through the whole. Hence it inflames, and ſuppurates. In order to prevent miſchief, Mr. HUNTER adviſes that the orifice ſhould be cloſed as accurately as poſſible ; and he recommends that the orifice in the ſkin ſhould be drawn to one ſide of that in the vein, ſo as to make the ſkin do the office of a valve to the venal orifice."

In Dr. VAUGHAN's caſe, that the inflammation affected the lymphatics, and that matter was abſorbed by thoſe lymphatics, cannot be doubted ; and I think there is no reaſon to doubt, that the matter was generated in the arm itſelf.

The man was bled on Sunday, and no pain was felt till Tueſday evening; when ſuppuration might not only be expected, but we are poſitively informed by Dr. V. that it had taken place, and was perceived by the ſurgeon. Dr. V's. account agrees with reaſon and experience, however erroneous his concluſion may be. He ſays, *the ſides of the vein did not appear to be united, but inflamed and ſuppurated* : and again, that *the wound of the vein underwent inflammation and ſuppuration,* and *then* the diſeaſe appeared.

He ſays, " Its appearance ſo ſoon after the application of the poiſon was probably owing to Mr. GREEN being naturally *a healthy man* ; and to his labouring under *no other diſeaſe* at the ſame time."—This remark alone is fatal to Mr. P's. hypotheſis.

But ſo far from the time being remarkably ſhort, for ſo formidable a diſeaſe to be occaſioned by inflammation, ſuppuration, and abſorption of matter ; it is not a fortnight ſince I was called to a lady, in whoſe arm a conſiderable number of lymphatics, highly inflamed, might be traced up to the axilla, attended with violent pain. The cauſe was a

ſmall

ſmall whitlow, of no long ſtanding, on the thumb. By opening it, removing a ſmall portion of the ſkin, enſuring a free diſcharge, and cutting off the ſupply of matter; and, perhaps, full as much, by taking off tenſion and irritation by the means above mentioned, and the application of a poultice to the abſceſs, and cold water to the arm, all pain and miſchief were ſoon removed. Similar ill effects, ariſing from confined matter in every part of the hand and arm, are too common, and too well known to every ſurgeon, to require a minute recital.

Theſe remarks may to ſome readers appear ſuperfluous; if they appear ſo to you, I beg you will ſuppreſs them; if not, you will oblige me by inſerting them in the next Number of your reſpectable Journal.

I am, Gentlemen,

New Street, Hanover Square, Your obedient ſervant,

